# Septic arthritis with osteomyelitis due to *Salmonella enterica* serotype Dublin: A case series

**DOI:** 10.3389/fsurg.2022.1069141

**Published:** 2023-01-06

**Authors:** Boyi Jiang, Hong Xu, Zongke Zhou

**Affiliations:** Department of Orthopaedic Surgery, West China Hospital, Sichuan University, Chengdu, China

**Keywords:** septic arthritis, osteomyelitis, debridement, twostage exchange, salmonella dublin

## Abstract

**Background:**

Septic arthritis with osteomyelitis due to *Salmonella enterica* serotype Dublin is rare. We reviewed and analyzed cases of septic arthritis with osteomyelitis due to *Salmonella enterica* serotype Dublin seen at our institution.

**Methods:**

The medical records of all patients diagnosed with Salmonella septic arthritis and/or Salmonella osteomyelitis during 2017–2022 were included. We reviewed the diagnosis, medical history, clinical management, and outcome of all cases.

**Results:**

Five patients with *Salmonella* septic arthritis or *Salmonella* osteomyelitis were identified during the 5-year study period. They were all male; the median age was 53 years (range 15–56). Only one was immunodeficient. All five patients were infected at the hip joint and ipsilateral femur, while two suffered bilateral hip septic arthritis with femoral osteomyelitis. *Salmonella* Dublin was isolated from the hip joint fluid of all patients. Four presented with fever and constitutional signs within four weeks of symptom onset. Four had positive blood cultures, and only one patient had gastrointestinal symptoms. Four patients underwent surgical debridement as the primary surgical plan, and two underwent secondary two-stage exchange after primary surgical debridement failure. The last patient had a two-stage exchange directly as the first surgical treatment. All patients received intravenous antimicrobial therapy for a median duration of 6 (range 4–12) weeks and oral antimicrobial therapy for a median duration of 4 (range 4–6) weeks. All patients had a median duration of follow-up of 12 months (range 9–25), and none had evidence of recurrence of infection.

**Conclusions:**

Septic arthritis due to *Salmonella* Dublin remains rare. It frequently occurs with ipsilateral femur osteomyelitis adjacent to the infected hip joint in our cases. Surgical debridement or two-stage exchange, along with 4–12 weeks of effective intravenous and followed by 4–6 oral antimicrobial therapy, could successfully eradicate the infection.

## Introduction

Septic arthritis is the most rapidly destructive joint disease; it is relatively rare in the general population, and its yearly incidence varies from 4 to 10 per 100,000 in Western Europe to 29 cases per 100,000 in disadvantaged groups from Northern Europe and Australia; however, its mortality rate due to its complications is as high as 11% (1, 2). Moreover, cured patients often suffer from varying degrees of joint dysfunction (2). The most frequent causative organisms are gram-positive bacteria, such as Staphylococcus aureus (1).

*Salmonella* is one of the Gram-negative bacteria, of which nontyphoidal *Salmonella* (NTS) is a common pathogen that causes human foodborne infections (3). NTS infection of joints and bones is rarer, accounting for only 0.8% of all *Salmonella* infections and 0.45% of all types of osteomyelitis, usually occurring in patients with underlying medical conditions, such as sickle cell disease, systemic lupus erythematosus (SLE), immunosuppression, diabetes, etc (4–7). *Salmonella enterica* Serotype Dublin (*S.* Dublin) is a host-adapted serotype to cattle (8, 9). Studies have shown that *S.* Dublin is particularly aggressive in humans, more likely to cause bloodstream infections, and more often lead to severe disease and higher rates of antimicrobial resistance than other serotypes (9, 10). Most of the human risk of infection with *Salmonella* Dublin is caused by the consumption of raw milk, milk products and raw beef (10, 11). With the increased consumption of these foods worldwide, there have been only infrequent case reports of septic arthritis or osteomyelitis caused by *Salmonella* Dublin in this time period. Being a rare and reportable disease, and its management protocols and outcomes are also not well defined, we focus on exploring these similar diseases and strive to provide early and effective therapeutic management.

We therefore retrospectively reviewed all patients with a diagnosis of septic arthritis and/or osteomyelitis caused by *Salmonella* Dublin seen at our institution during 2017–2022 and evaluated the demographics, clinical manifestations, treatment and outcomes of these infections.

## Methods

### Study design

This is a single-center retrospective case series undertaken at the West China Hospital of Sichuan University. Our study design was approved and was considered exempt by the Ethics Committee on Biomedical Research, West China Hospital of Sichuan University (approval no. 2022-1494). All patients provided informed consent.

### Case ascertainment and data collection

Study patients were evaluated at our institution between 9/1/2017 and 9/1/2022. Cases were ascertained by searching our institution's medical and surgical indices and the microbiology database. Patients who were diagnosed with septic arthritis and/or osteomyelitis due to *Salmonella* Dublin were included. Information was available for all patients. Patients were followed until the development of surgical treatment failure, death or loss to follow-up. Descriptive statistics were used to summarize the demographic, clinical and surgical treatment details and were analyzed using IBM SPSS version 21 software (IBM Corp., Armonk, NY, United States).

### Definitions

We employed the Newman criteria (12) to diagnose septic arthritis. These criteria require 1 of 4 criteria to be met to consider a diagnosis of septic arthritis: (1) Isolation of an organism from an affected joint. (2) Isolation of an organism from another source with an associated hot and swollen joint. (3) Joint pain and swelling and turbid joint fluid in the presence of previous antibiotic therapy. (4) Histologic or radiologic evidence consistent with septic arthritis (1).

Pyogenic osteomyelitis was diagnosed if the following criteria were met (13): (a) clinical presentation consistent with a bone infection (fever, local soft tissue swelling, bone pain and abnormal physical examination); and/or (b) positive blood or puncture fluid culture or positive bone biopsy.; and/or (c) presence of radiological signs consistent with osteomyelitis [especially magnetic resonance imaging (MRI) scan]; and/or (d) surgical finding of pus in the affected marrow cavity.

*Salmonella* septic arthritis or osteomyelitis was diagnosed if it met the above criteria and if *Salmonella* species were isolated from two cultures of joint/bone aspirates or intraoperative tissue specimens, purulence in the affected joint/marrow cavity at the time of surgery with one positive culture yielding *Salmonella* species.

Patients were either classified as having a good outcome or having failed treatment. Treatment failure was defined by one of the following criteria: recurrence of septic arthritis or osteomyelitis due to the same Salmonella strain; death due to septic arthritis or osteomyelitis-related infection and indeterminate clinical failure, defined as clinical, laboratory, or radiological findings suggestive of septic arthritis or osteomyelitis at any time after initial therapy. Patients who did not fulfill the criteria for treatment failure were characterized as having a “good outcome”.

## Results

### Patient cohort

In our cohort of five patients, the median age at diagnosis of *Salmonella* septic arthritis with osteomyelitis in our hospital was 53 years (range 15–56). All five patients were male and were diagnosed within the past three years. A summary of the five patients is presented in Table 1.

**Table 1:** Summary of the 5 patients with 10 episodes of *Salmonella* septic arthritis with osteomyelitis.

Patient no.	Infected joints/bones	Comorbidities	Immunodeficient	Gastrointestinal symptoms	Bacteremia	Clinical presentation	Microbiology	Antimicrobial therapy	Surgial management	Failed treatment	Follow-up duration
1	right hip	–	–	–	yes	Joint pain and dysfunction × 7 days and fever × 1 day	Salmonella enterica Serotype Dublin (aspirate joint fluid and blood culture)	IV imipenem-cilastatin sodium combined vancomycin× 11 days	joint debridement, irrigation and drainage	yes	11 days
	right femur				yes	recurrent fever, right thigh pain, MRI suggests femoral osteomyelitis	"(operative sample)	IV ceftriaxone × 4 weeks and oral TMP-SMX × 4 weeks	femur debridement and decompression	no	12 months
2	bilateral hip	–	–	–	yes	Joint pain and dysfunction, intermittent fever × 4 weeks	Salmonella enterica Serotype Dublin (aspirate joint fluid and blood culture)	IV Piperacillin-tazobactam × 4 weeks and vancomycin × 10 days	joint debridement, irrigation and drainage	yes	13 days
	bilateral femur				yes	recurrent fever, bilateral thigh pain, MRI suggests femoral osteomyelitis	"(operative sample)	IV imipenem-cilastatin sodium combined linezolid × 12 weeks and oral ciprofloxacin × 6 weeks	femur debridement, decompression and drainage	no	10 months
3	left hip	CKD stage 4, DM, bilateral ONFH	now on methylprednisolone and mycophenolate mofetil	–	no	Deep joint pain, discomfort × 8 months	Salmonella enterica Serotype Dublin (aspirate joint fluid)	IV moxifloxacin combined ceftazidime × 5 weeks and oral ceftazidime × 8 weeks	joint debridement, irrigation and drainage	yes	7 months
	left hip and femur				no	Joint dysfunction × 7 months	"(operative sample)	IV ceftriaxone × 5 weeks and oral TMP-SMX combined cefaclor ×4 weeks	Two- stage exchange treatment	no	12 months
4	left hip	–	–	–	yes	Joint pain and dysfunction × 1 week	Salmonella enterica Serotype Dublin (aspirate joint fluid)	IV ceftriaxone × 6 weeks	joint debridement, irrigation and drainage (twice in other hospital)	yes	8 months
	left hip and femur				no	Joint pain and dysfunction × 2 weeks and fever × 1 week	"(operative sample)	IV imipenem-cilastatin sodium × 4 weeks combined vancomycin × 3 weeks and oral ciprofloxacin combined rifampicin × 4 weeks	Two- stage exchange treatment	no	25 months
5	bilateral hip	–	–	diarrhea for two months	yes	Deep joint and thign pain, discomfort × 1 week	Salmonella enterica Serotype Dublin (aspirate joint fluid)	IV ceftriaxone × 8 weeks	-	yes	6 months
	bilateral hip and femur			–	no	Joint pain and dysfunction × 1 weeks	"(operative sample)	IV imipenem-cilastatin sodium combined vancomycin × 6 weeks and oral ciprofloxacin × 6 weeks	Two- stage exchange treatment	no	12 months

TMP-SMX, trimethoprim-sulfamethoxazole; CKD, chronic kidney disease; DM, diabetes mellitus; ONFH, osteonecrosis of the femoral head.

All five patients were infected at the hip joint and ipsilateral femur, while two of them also suffered bilateral hip septic arthritis with femoral osteomyelitis. *Salmonella* Dublin was isolated from the hip joint fluid of all five patients. No patient had a history of prior septic arthritis or osteomyelitis on the same or different joints and bones. Only one patient suffered from CKD stage 4 requiring long-term oral methylprednisolone and mycophenolate mofetil. This patient's other comorbidities included diabetes mellitus and bilateral osteonecrosis of the femoral head (ONFH). The remaining four patients were in normal immune status and had no underlying comorbidities.

Patients 1 and 2 presented acutely with signs and symptoms present for less than four weeks and were febrile at presentation. Patients 3, 4 and 5 presented with chronic symptoms when coming into our hospital (6–8 months of deep hip pain). The median duration of chronic joint symptoms prior to diagnosis was 7 (range 6–8) months. All five patients initially presented with pain in the involved hip and gradually developed pain in the ipsilateral thigh over the course of the disease. Patient 4 developed a sinus tract adjacent to his left hip incision scar after undergoing two debridement failures outside our hospital.

Patient 5 had a history of eating sashimi, followed by diarrhea in the preceding two months before symptom onset, but he had a negative stool culture when he came to our hospital. Four patients had documented *Salmonella* bacteremia (positive blood cultures) before hip septic arthritis with femoral osteomyelitis was diagnosed.

### Diagnosis

All first episodes of *Salmonella* septic arthritis or osteomyelitis were diagnosed in accordance with the definitions described in the methodology section. The five patients we reported all had *Salmonella enterica* Serotype Dublin. All isolates were nonsusceptible to first-generation cephalosporin macrolides, aminoglycosides and nalidixic acid. One of them was nonsusceptible to ciprofloxacin, and the others were susceptible to ciprofloxacin.

The median erythrocyte sedimentation rate (ESR), C-reactive protein (CRP) values and interleukin-6 (IL-6) values at presentation were 79 mm/1st hour (range 62–120), 79.3 mg/L (range 6.56–306) and 29.4 pg/ml (range 3.74–282.8), respectively (normal range of ESR 0–21 mm/1 h, CRP <5 mg/L and IL-6 <7 pg/ml). White blood cell counts (WBCs) and neutrophilic granulocyte counts (NEUTs) were within normal limits for each patient on admission. The median WBC and NEUT were 7.34 × 10^9^/L (range 6.51–9.25) and 5.42 × 10^9^/L (range 3.23–6.29), respectively (normal range of WBC 3.5–9.5 × 10^9^/L and NEUT 1.8–6.3 × 10^9^/L). Every patient had available pathology reports; two were confirmed to have acute inflammation manifested as congestion, edema, and massive neutrophil infiltration, while the other three had fibroreactive changes, necrosis and perinecrotic inflammatory cell infiltration without evidence of acute inflammation.

### Management and outcome

Clinical management included antimicrobial therapy only, surgical debridement, and two-stage exchange.

Patients 1 and 2 presented with acute septic arthritis. Patient 1 underwent direct hip debridement, catheter irrigation and drainage, and patient 2 underwent bilateral hip debridement, catheter irrigation and drainage after failing four weeks of intravenous antibiotics. As the disease progressed, both patients were found to also co-exist with ipsilateral Salmonella osteomyelitis of the femur and underwent debridement and decompression of the ipsilateral femur.

**Figure 1:**
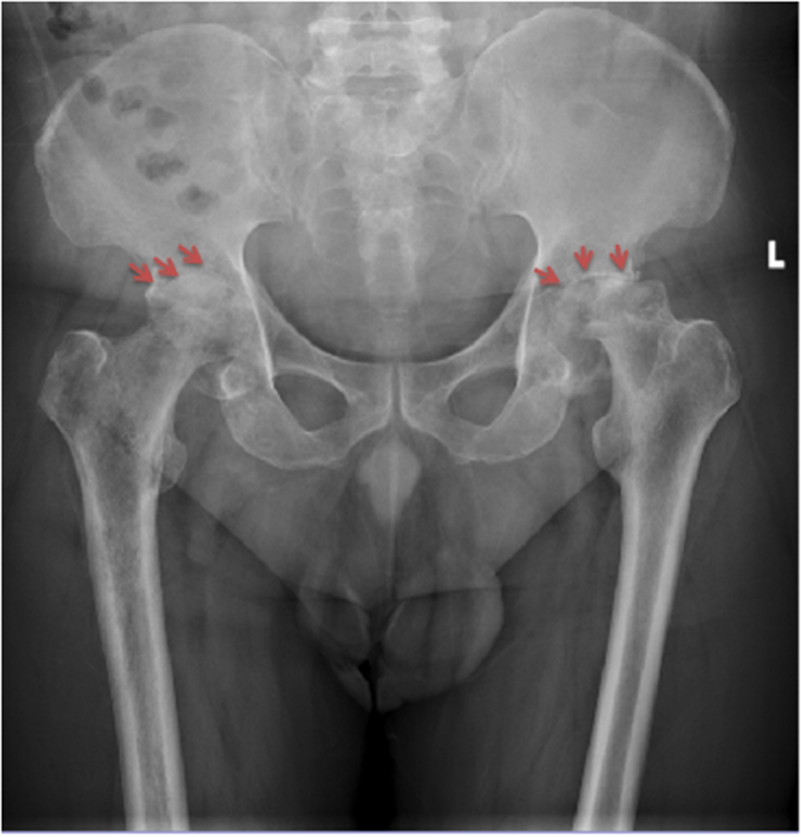
Patient 5, a 52-year-old male, anteroposterior radiograph of the hip revealed severe joint space narrowing, disappearance of the acetabular sourcil, and bone destruction in the femoral head and femoral neck. Images were taken six months after the onset of hip pain and fever.

**Figure 2:**
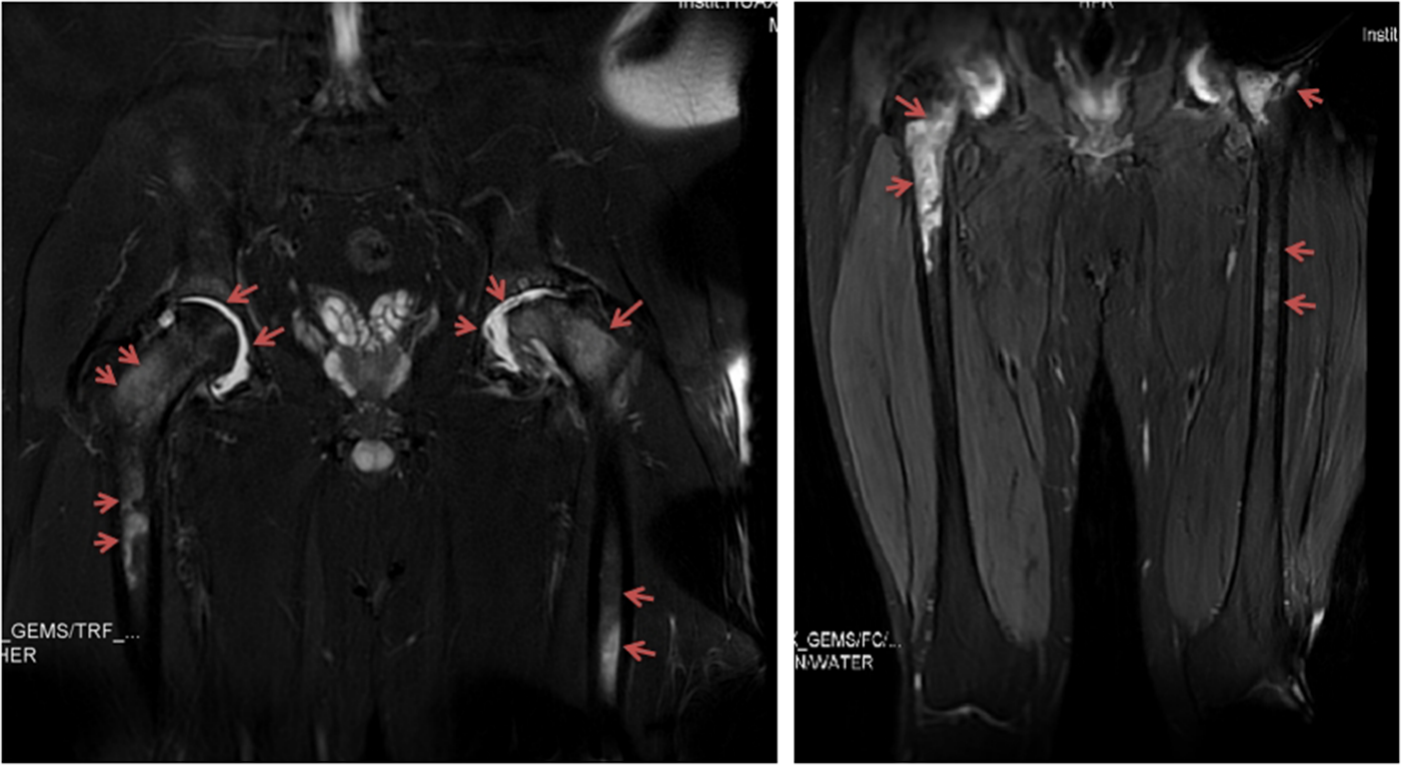
Patient 5: magnetic resonance imaging of the hip joint indicated joint effusion and periarticular bone and femur marrow signal changes. Images were taken six months after the onset of hip pain and fever.

**Figure 3:**
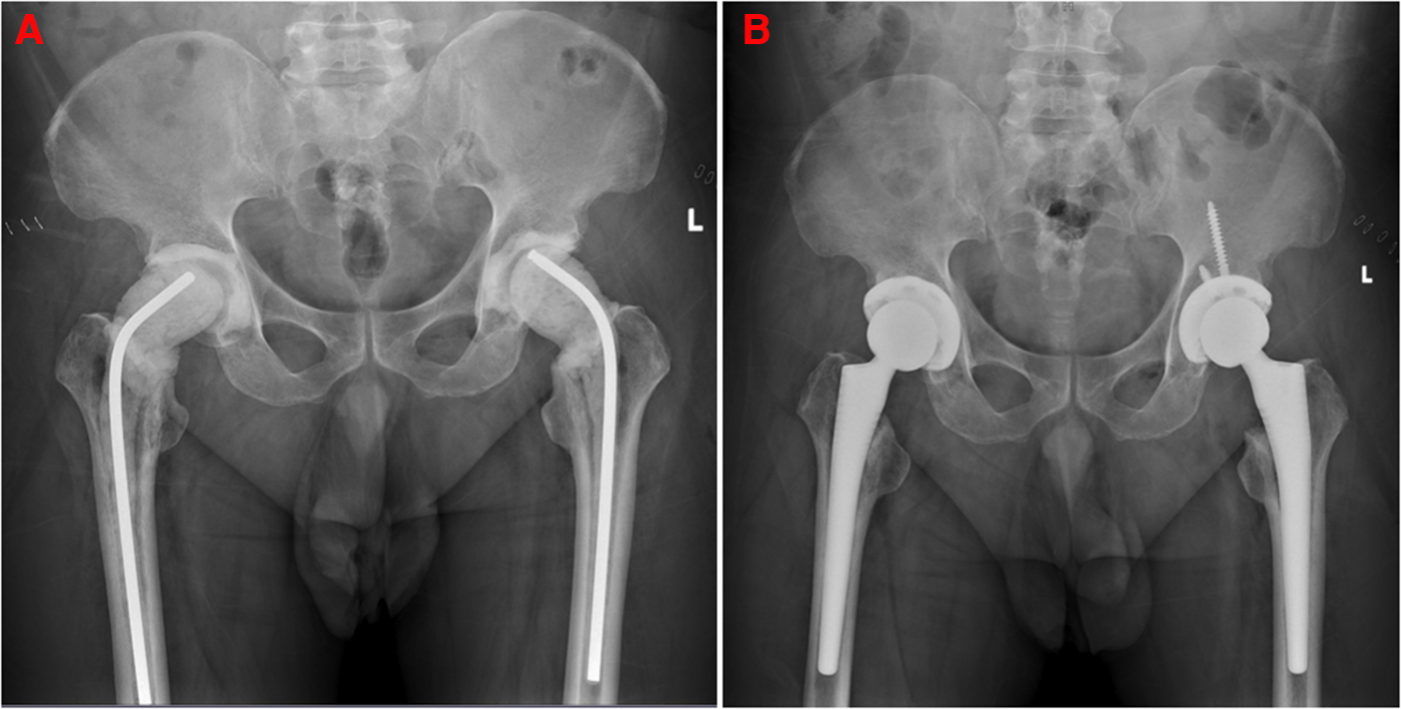
(**A**) Patient 5, anteroposterior radiograph of the hip taken after first-stage surgery. (**B**) shows an anteroposterior radiograph of the hip taken six months after stage 2 bilateral THA, indicating the prosthesis in place and good bone ingrowth without osteolysis.

Patients 3, 4 and 5 presented with chronic septic arthritis. Patient 3 initially underwent debridement and antibiotic treatment of the affected hip in our hospital and was readmitted for two-stage exchange after the prior debridement failure. Patient 4 underwent two-stage exchange in our hospital after failing two hip debridements outside. Patient 5 underwent two-stage exchange in our hospital after failing conservative treatment with antibiotics for 8 weeks outside (Figures 1–3).

In total, two cases underwent surgical debridement only, and three required final two-stage exchange to eradicate the infection. The first stage entails debridement of the infected tissues of the hip, resection arthroplasty of the proximal femur followed by implantation of an antibiotic-loaded (vancomycin and tigecycline) cement spacer. The second stage involved implantation of a total hip replacement when satisfactory infection control was achieved. The standard of infection control is wound healing and normal erythrocyte sedimentation rate/C-reactive protein level measured at three consecutive follow-up visits, negative clinical signs and symptoms, negative radiological findings, and a negative joint fluid culture (14). All patients received intravenous antimicrobial therapy for a median duration of 6 (range 4–12) weeks and oral antimicrobial therapy for a median duration of 4 (range 4–6) weeks. All patients had a median duration of follow-up of 12 months (range 9–25), and none had evidence of recurrence of infection. The median Pain VAS and Harris hip score of patients were 8 points (range 7–9) and 20 points (range 11–23) respectively at admission. One year after surgery, all patients' pain level was greatly reduced and hip function was significantly improved. The median Pain VAS and Harris hip score were 1 point (range 1–2) and 87 points (range 59–88) respectively. There were significant differences in the two scores before and after the treatment (Table 2).

**Table 2:** Summary and comparison of the VAS pain score and Harris hip score of 5 patients at admission and one year after surgery.

Patient no.	Infected joints	Pain VAS			Harris hip score		
		at admission	one year after surgery	*P*-value	at admission	one year after surgery	*P*-value
1	right hip	8	1	.000*	15	65	.000*
2	right hip	9	2		11	59	
	left hip	9	2		11	61	
3	left hip	7	1		20	88	
4	left hip	8	1		23	87	
5	right hip	7	1		21	87	
	left hip	7	1		21	87	

Pain VAS: 0 = no pain, 1–3 = mild pain, 4–6 = moderate pain (45–74 mm), and 7–10 = severe pain; Harris hip score: <70 = poor result, 70–80 = fair, 80–90 = good, and 90–100 = excellent.

* *P* < 0.05.

## Discussion

Our study showed that septic arthritis due to *Salmonella* Dublin remains rare, and this serotype may affect bones and joints through bloodstream infection, with fewer gastrointestinal symptoms. In our cases, septic arthritis frequently occurred with osteomyelitis adjacent to the infected joint, and most were immunocompetent adults, which also suggested that *S.* Dublin is invasive. All patients required surgery combined with sensitive antibiotics to achieve better outcomes.

*Salmonella* infection is a public health problem worldwide and accounts for at least one-third of all outbreaks in the United States (3, 15). Septic arthritis is an uncommon consequence of NTS infection, with an estimated incidence of less than 0.1%–0.2% (16). Jones et al. revealed 46,639 positive cultures for *Salmonella*; 5% of those cases were isolated from blood and only 0.06% from bone or joint synovial fluid (17). The pathway by which *Salmonella* causes septic arthritis is generally considered to be hematogenous (18) and occurs more often in patients with immunodeficiency or underlying diseases (4, 5, 19, 20). Bone and joint infections due to *Salmonella* in immunocompetent adults are very rare. However, disease severity varies widely between different *Salmonella* serotypes; Salmonella Dublin is known to be more likely than other NTS species to cause serious problems, such as bloodstream infections (17, 21), and the majority do not have immunodeficiency. In our case, four patients were immunocompetent and presented with joint pain, fever exceeding 38.5°C, and blood cultures found *S.* Dublin (with the same species identified in synovial fluid and operative cultures). The only patient with a negative blood culture suffered from CKD stage 4 requiring long-term oral immunosuppressive drugs. The study found that the mean age of infection with *Salmonella* Dublin is 54 years (17). Harvey et al. also reported that 60% of *Salmonella* Dublin infections occurred in men (10). Our case review is consistent with previous reports, in which all were males and the median age of infection was 53 years. Previous studies have confirmed that S. Dublin is frequently isolated from live cattle and that raw unpasteurized milk or cheeses made from milk may be contaminated with *S.* Dublin (10, 11). Many studies have found that men consume more undercooked beef than women (22, 23). Occupational exposure to cattle may also lead to an increased frequency of infection in males (10). However, our patient's occupation was not related to cattle, and there was no obvious history of exposure to raw beef or dairy products. We speculated that the initial gastrointestinal salmonellosis may be mild and may be overlooked. Of course, this may also be caused by a long incubation period and parenteral entry route of infection (24, 25).

The *S.* Dublin serotype has a higher proportion of resistant strains than other serotypes, according to the National Antimicrobial Resistance Surveillance System (NARMS) surveillance data (10). Harvey et al. showed that in 102 clinical isolates of *Salmonella* Dublin, 41% were pansusceptible, 55% were multidrug resistant, and among these MDR isolates, 84% were resistant to >5 classes of antimicrobials (10). In our case, the clinical isolates were resistant to first-generation cephalosporins, aminoglycosides, penicillins and nalidixic acid. Fortunately, only one isolate was resistant to ciprofloxacin but remained susceptible to third-generation cephalosporins. Both third-generation cephalosporins and fluoroquinolones have been successfully used in cases of *Salmonella* infection (6, 24). Fluoroquinolones are advocated as excellent choice drugs because of their potent anti-*Salmonella* activity, good bone penetration and ability to kill stationary phase as well as active phase bacteria (26). In our case review, ceftriaxone, ciprofloxacin, and more broad-spectrum and potent antibacterial drugs, such as carbapenems (imipenem-cilastatin), were the choices for septic arthritis due to *S.* Dublin. Broad-spectrum antibiotics such as carbapenems are used in some cases because patients still have recurrent bacteremia and fever despite the intravenous infusion of third-generation cephalosporin or fluoroquinolone. In addition, due to the symptoms of high fever, we added vancomycin to prevent possible concomitant gram-positive infection (6, 26).

Septic arthritis, an urgent disease, may damage cartilage directly through bacterial enterotoxins or indirectly due to the host's immune response to bacteria, requiring surgical intervention in most patients (27, 28). *Salmonella* often affects a single joint, and the hip joint is the most commonly infected joint (29). Our cases were all infected at the hip joint, and two were affected with bilateral hips. Surgical debridement is the first choice for septic arthritis to eradicate the infection (30). Routine open debridement includes removal of all accessible synovial tissue, capsulectomy, and drain placement (14). Drainage placement is fundamental to reduce intraarticular pressure and to minimize joint cartilage destruction. In our cases, two patients were diagnosed with acute septic arthritis, and preoperative MRI revealed significant articular surface damage; thus, we promptly performed surgical debridement in open procedures. However, they were subsequently found to combine with ipsilateral femoral osteomyelitis. The diagnosis of *Salmonella* osteomyelitis may be delayed because of insidious and overshadowed early symptoms by severe joint pain. After thorough joint debridement, they still had recurrent fever and began to describe thigh pain. The thigh MRI showed femoral osteomyelitis. Therefore, we performed femoral debridement, decompression, and drainage. Their symptoms were eventually relieved, with no evidence of recurrence of infection. For this type of coexisting and insidious Salmonella osteomyelitis, we recommend performing MRI scan on adjacent areas of the infected joint to identify and minimize harm to patients in early clinical management. The prognosis of septic arthritis after *Salmonella* infection is unclear; although most patients show resolution of arthritis within four months of onset, chronic symptoms can persist for up to five years (3). Chronic hip infection may gradually lead to joint degeneration, complete joint destruction or even result in chronic osteomyelitis of the proximal femur. If in this situation, we recommend a two-stage exchange. Thorough joint and medullary debridement and cement-loaded spacers can effectively eradicate infection and improve pain (31–33). Then, the second-stage THA can further restore good hip motion. In our case, three patients were diagnosed with chronic septic arthritis with femoral osteomyelitis. They had long-term joint pain and dysfunction and even needed a walker or wheelchair to maintain their normal life. X-ray and MRI showed severe joint destruction, joint space narrowing, and erosion of the proximal femur. Considering the above situation and that they were all over 50 years old, we performed two-stage exchange surgery and achieved satisfactory outcomes.

Success rates described in the literature may vary due to limited follow-up time, different definitions of successful outcomes, and the propensity for publication bias of successfully treated case reports. In our cohort, the median duration of follow-up was twelve months. There are several limitations to this study. First, this study was retrospective and had the same biases inherent in other retrospective studies. Second, the limited sample size is a function of the low incidence of the disease. Third, all of our cases occurred within the past three years; thus, the follow-up time was relatively short, and the long-term clinical outcome could not be determined. Nevertheless, this series of septic arthritis with osteomyelitis due to *Salmonella* Dublin supports the value of early joint debridement or two-stage exchange combined with susceptible antibiotic therapy in these patients.

## Conclusions

Septic arthritis due to *Salmonella* Dublin remains rare, but this serotype is more invasive and more frequently infects immunocompetent adults. The presentation is often acute with fever, local signs and elevated inflammatory biomarkers. It frequently occurs with ipsilateral femur osteomyelitis adjacent to the infected hip joint in our cases. Treatment requires an early surgical approach, such as surgical debridement or two-stage exchange, combined with 4–12 weeks of effective intravenous and followed by 4–6 oral susceptible antibiotic drug therapy was most often associated with successful eradication of infection.

## Data Availability

The original contributions presented in the study are included in the article/Supplementary Material, further inquiries can be directed to the corresponding author/s.
